# Synergistically Modified CNF/PAM Hydrogel with LiCl and Carboxylated Graphene for Zinc–Air Batteries

**DOI:** 10.3390/polym18182243

**Published:** 2026-09-15

**Authors:** Weiyue Liu, Mingxuan Liu, Luqing Zhang, Sitong Guo, Yifeng Ruan, Ruiwen Wang, Chenhui Yang

**Affiliations:** 1 State Key Laboratory of Utilization of Woody Oil Resource, Northeast Forestry University, Harbin 150040, China; 2023220496@nefu.edu.cn (W.L.); liumx@nefu.edu.cn (M.L.); 17535714662@163.com (L.Z.); gst826293283@163.com (S.G.); 18745447299@163.com (Y.R.); wrwnefu@163.com (R.W.); 2 Key Laboratory of Bio-Based Material Science & Technology of Ministry of Education, Northeast Forestry University, Harbin 150040, China

**Keywords:** zinc–air battery, flexible hydrogel electrolyte, dual-crosslinked network, cellulose nanofibers, carboxylated graphene, lithium chloride

## Abstract

Flexible zinc–air batteries (ZABs) with high theoretical energy density, intrinsic safety, and low cost have gained significant attention for flexible energy storage devices. However, conventional single-component electrolytes struggle to simultaneously meet comprehensive performance requirements, including mechanical strength, conductivity, practical specific capacity, and cycling stability. Herein, carboxylated graphene (CG) and lithium chloride (LiCl) were incorporated into a cellulose nanofibril/polyacrylamide (CNF/PAM) hydrogel matrix to fabricate a series of multi-component, dual-crosslinked high-performance composite hydrogel electrolytes toward flexible and rechargeable zinc–air batteries. The results reveal that the LiCl-CG@CNF/PAM composite hydrogel achieves an ionic conductivity of up to 4.473 mS·cm^−1^ and exhibits excellent mechanical properties, with a tensile elongation of 457% at a stress of 151.5 kPa. The assembled flexible ZABs deliver a peak power density of 60.7 mW·cm^−2^, a steady discharge duration of 38 h, and a long cycling lifetime of 47 h. This hydrogel electrolyte affords a novel strategy for the design and fabrication of high-performance, long-lifetime flexible wearable zinc–air batteries.

## 1. Introduction

With the rapid proliferation of flexible wearable electronics, rechargeable zinc–air batteries (ZABs) have emerged as a premier option for next-generation energy storage [1,2,3,4,5] due to their high energy density, cost-effectiveness, and intrinsic safety. However, conventional liquid alkaline electrolytes are plagued by leakage, carbonation, and severe zinc anode corrosion/dendrite growth, which impede their practical utilization in wearable devices [6,7,8,9]. To eliminate these hazards, solid-state hydrogel electrolytes constructed from crosslinked polymer networks have garnered significant interest [10,11,12]. Nevertheless, conventional single-component synthetic hydrogels rarely meet the comprehensive requirements of high mechanical robustness, favorable ionic conductivity, and long-term electrochemical stability simultaneously, typically suffering from high concentration polarization or structural failure under demanding operating conditions [13].

To break this performance trade-off, integrating rigid biomass networks into synthetic polymer matrices has been widely explored. Cellulose nanofibers (CNFs), featuring high moduli, rich surface hydroxyls, and high aspect ratios, serve as an ideal skeleton to establish tough secondary physical networks [14]. However, a critical dilemma remains: the incorporation of nanocellulose dramatically increases local gel viscosity, which severely restricts oxygen mass transport to the air cathode during aggressive discharge [15]. This severe concentration polarization broadens the voltage gap and limits the battery’s peak power density. Therefore, implementing a multi-component synergistic modification strategy within the dual-network framework is essential to streamline both ion migration paths and gas transport microenvironments [16,17].

Tailoring the interfacial properties via functional fillers and inorganic salts provides a promising route to address these kinetics limitations. Compared to standard graphene oxide [18,19], carboxylated graphene (CG) possesses abundant active -COOH groups that afford superior aqueous dispersibility and strong interfacial compatibility with amide-rich polymers; meanwhile, it preserves intact sp^2^ carbon skeletons to construct continuous conductive channels, realizing efficient electron transport. Crucially, the highly active carboxyl groups on CG nanosheets exert an electrostatic shielding effect that homogenizes zinc-ion flux and suppresses dendritic proliferation. Concurrently, incorporating lithium chloride (LiCl) introduces small-sized Li^+^ ions with high mobility, which effectively suppresses ion pairing, reorganizes hydrated conduction pathways, and alleviates electrode passivation in concentrated systems [20,21,22]. Moreover, strong ion–dipole electrostatic interactions between Li^+^ and the polar functional groups within the gel function as reversible sacrificial bonds, significantly dissipating mechanical stress and stabilizing the solid–liquid boundary [23,24].

**Figure 1:**
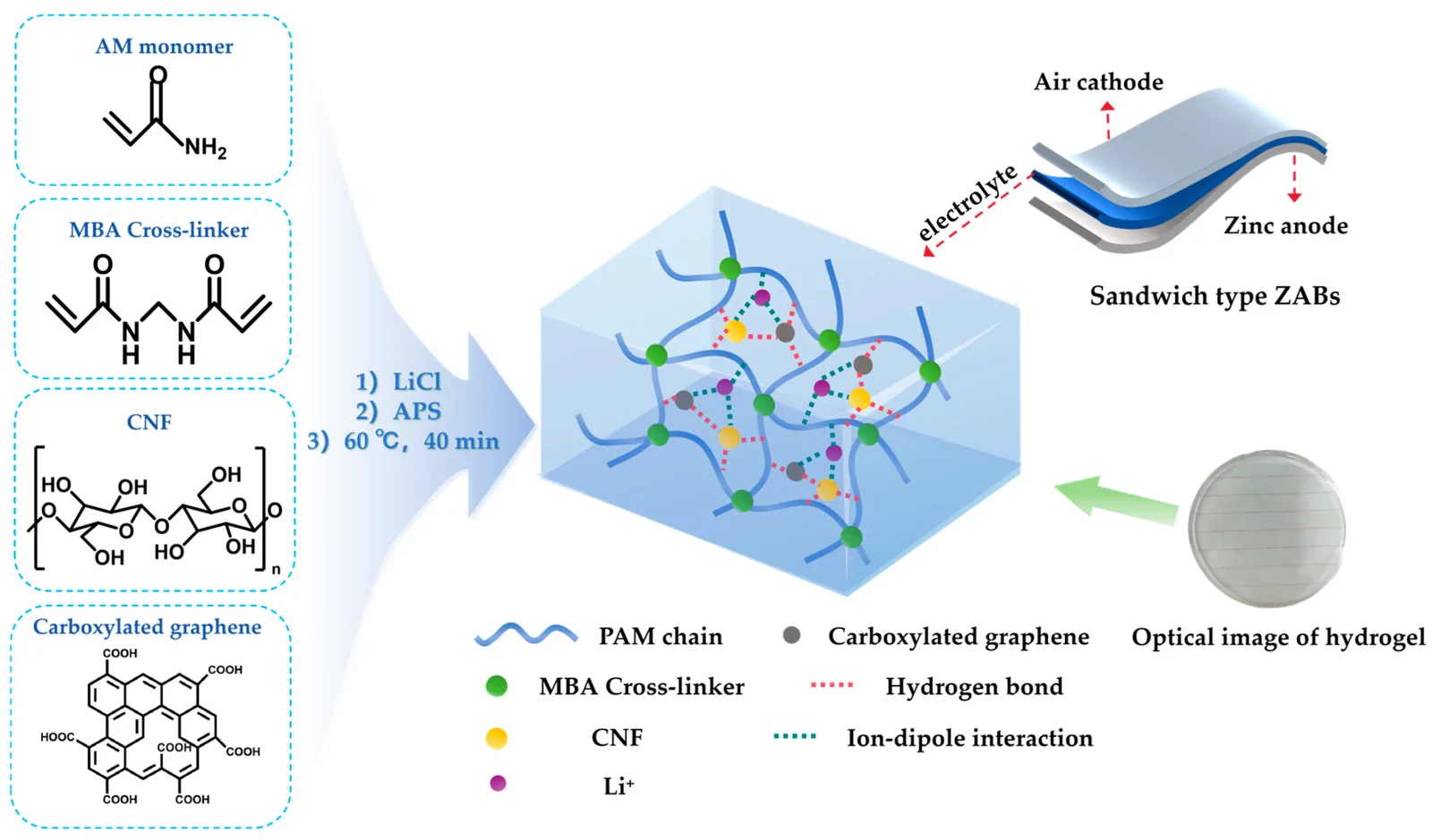
Fabrication process of LiCl-CG@CNF/PAM hydrogel.

Herein, we report a novel, high-performance, multi-component, dual-crosslinked composite hydrogel electrolyte (LiCl-CG@CNF/PAM) fabricated via a facile one-pot in situ free-radical polymerization strategy (Figure 1). The architecture features a covalently crosslinked PAM-MBA primary chemical network intertwined with a multi-layered secondary dynamic physical network stabilized by CNF-CG hydrogen bonding and Li^+^-mediated ion–dipole interactions. By integrating a biomass scaffold, carboxyl–carbon fillers, and ion-assisted crosslinkers, this novel combination is scarcely reported in current hydrogel electrolyte systems for zinc–air batteries. This advanced design successfully circumvents the classic trade-off between mechanical strength and ionic transport kinetics by optimizing the interconnected micro-morphology and reducing internal resistance. Consequently, the optimized LiCl_0.2_-CG_0.2_@CNF_2wt%_/PAM hydrogel achieves an elevated ionic conductivity of 4.473 mS·cm^−1^ and an outstanding tensile elongation of 457%. The assembled flexible quasi-solid-state ZABs deliver an impressive peak power density of 60.7 mW·cm^−2^ alongside an exceptional discharge duration of 38 h and a stable cycling lifespan of 47 h, offering a viable paradigm for durable wearable energy storage devices.

## 2. Materials and Methods

Acrylamide (AM, 99.0%), N,N′-methylene-bisacrylamide (MBA, 99.0%), ammonium persulfate (APS, ≥98.0%) and lithium chloride (LiCl, ≥99.0%) were acquired from Shanghai Aladdin Biochemical Technology Co., Ltd. in Shanghai, China. Cellulose nanofibers (CNF, 2.5 ± 0.5 wt%) were provided by Zhongshan Nanofiber New Materials Co., Ltd. in Zhongshan, China. Carboxylated graphene (CG, 2 mg·mL^−1^) was supplied by Jiangsu Xianfeng Nano Materials Technology Co., Ltd. in Nanjing, China. Potassium hydroxide (KOH, ≥85%) was obtained from Tianjin Tianli Chemical Reagents Co., Ltd. in Tianjin, China. Zinc acetate dihydrate (Zn(OAc)_2_·2H_2_O, ≥99.0%) was purchased from Tianjin Fuchen Chemical Reagents Plant in Tianjin, China. Zinc foil with a thickness of 0.2 mm and air electrodes consisting of nickel foam current collectors and Co_3_O_4_ catalysts were provided by Shanghai Yanwen Technology Co., Ltd. in Shanghai, China.

### 2.1. Synthesis of the Hydrogel Electrolyte

The hydrogel electrolyte was fabricated via a one-pot in situ free-radical polymerization strategy. First, CNF dispersions with mass fractions of 1, 2 and 3 wt% were prepared by homogenization using an ultrasonic cell grinder (JY92-IIN, Ningbo Xinzhi Biotech Co., Ltd. in Ningbo, China). Separately, 6.8 g AM and 12.1 mg MBA were dissolved in 13.2 g deionized water under stirring for 10 min to yield the AM/MBA solution. Then, 10.0 mL of the prepared CNF dispersion was added to the AM/MBA solution, followed by stirring for 5 min to obtain a uniform CNF/PAM precursor. Subsequently, 3.6 mg APS was added as an initiator; the mixture was subjected to magnetic stirring and ultrasonication for 20 min to ensure uniform dispersion. Finally, the resulting mixture was poured into a Petri dish and thermally polymerized at 60 °C for 40 min in a constant-temperature drying oven (DZF-6050, Shanghai Yiheng Scientific Instrument Co., Ltd. in Shanghai, China), yielding CNF/PAM composite hydrogels with different CNF loadings.

For multi-component LiCl-CG@CNF/PAM hydrogels, the optimal CNF_2wt%_/PAM formulation was adopted as the matrix. CG dispersion (2 mg·mL^−1^) was added to the CNF/PAM precursor in volumes of 0.10, 0.15, and 0.20 mL and sonicated for 5 min. Afterwards, LiCl aqueous solution (2 mg·mL^−1^) with corresponding volumes of 0.10 mL, 0.15 mL, and 0.20 mL was added and thoroughly blended. The mixtures were poured and thermally polymerized under the same conditions as described above. CG@CNF/PAM hydrogels were prepared following the same procedure but omitting LiCl. A series of composite hydrogels denoted as CGx@CNFy/PAM and LiClx-CGx@CNFy/PAM were fabricated. In these labels, parameter x (0.1, 0.15, 0.2, 0.3, 0.4) refers to the volume (mL) of CG stock solution (2 mg mL^−1^) added to the precursor mixture. Parameter y corresponds to the mass fraction of CNF in the hydrogel precursor, with values of 1 wt%, 2 wt%, and 3 wt%, respectively. The numbers following LiCl denote the feeding volume (mL) of LiCl solution. It is worth noting that all numerical values in the sample labels are feeding-related process parameters, rather than the actual mass fractions of the respective components in the cured hydrogel.

### 2.2. Characterization of the Hydrogel Electrolyte

Prior to characterization, all hydrogel samples were swollen in deionized water for 12 h and then freeze-dried for 48 h. Chemical functional groups were analyzed by Fourier transform infrared spectroscopy (FT-IR, Spectrum 3, PerkinElmer, Waltham, MA, USA) with attenuated total reflectance (ATR) in the range of 500–4000 cm^−1^. The crystal structure of the samples was characterized by X-ray diffraction (XRD, SmartLab SE, Rigaku, Akishima, Japan) with Cu Kα radiation. The scanning range was set from 5° to 80° (2θ) at a scanning rate of 2°/min. X-ray photoelectron spectroscopy (XPS, K-Alpha, Thermo Scientific, East Grinstead, UK) was used to analyze the surface elemental composition and chemical states. Morphological analysis was performed by scanning electron microscopy (SEM, Sigma 300, Carl Zeiss, Oberkochen, Germany).

Tensile properties were measured on a universal testing machine at a constant rate of 50 mm·min^−1^. Ionic conductivity was evaluated by electrochemical impedance spectroscopy (EIS) using an electrochemical workstation in AC impedance mode over a frequency range from 10^−2^ to 10^6^ Hz. For conductivity measurements, the hydrogel electrolyte was sandwiched between two copper electrodes; the ionic conductivity (*σ*) was calculated as

$$\sigma =\frac{1}{S_{0}\times R_{S}}$$

where l is the interelectrode distance (cm), *S*_0_ is the contact area between the electrolyte and the electrodes (cm^2^), and *Rs* corresponds to the high-frequency ohmic resistance derived from the Nyquist plot (Ω).

### 2.3. Assembly and Electrochemical Performance of Zn–Air Batteries (ZABs)

Flexible ZABs were assembled via a layer-by-layer sandwich configuration. Prior to assembly, the hydrogel electrolyte was soaked in an aqueous electrolyte of 6.0 M KOH and 0.2 M ZnSO_4_ for 24 h to ensure thorough impregnation. The pretreated hydrogel electrolyte was then sandwiched between a polished zinc foil anode and an air cathode comprising Co_3_O_4_ catalyst on nickel foam. The completed devices were sealed and fixed using porous plastic films.

Discharge polarization curves were implemented on the electrochemical workstation. The power density (*P*) was calculated as

P=V×I

where *V* is the discharge voltage (V) and *I* is the discharge current density (mA·cm^−2^).

Rate performance was evaluated at current densities of 0.1, 1, 2, 5, 10, 5, 2 and 1 mA·cm^−2^. A constant-current discharge curve at 2 mA·cm^−2^ was carried out to determine practical capacity and long-term cycling stability. Galvanostatic discharge and cycling performance were acquired with a LAND CT3001A battery test system (Wuhan LAND Electronics Co., Ltd. in Wuhan, China).

## 3. Results

### 3.1. Characterization of Chemical Composition and Microstructure

During in situ free-radical polymerization, AM is covalently crosslinked by MBA to form the primary chemical network. Concurrently, hydrogen bonds form among PAM amide groups, CNF hydroxyls and CG carboxyls, yielding a secondary physical network. Li^+^ ions interact with polar hydroxyl and amide groups via ion–dipole interactions [25]. The synergistic effect of covalent crosslinks, hydrogen bonds and ion–dipole interactions establishes a dual-crosslinked, porous microframework that enhances both mechanical properties and ionic transport [26].

**Figure 2:**
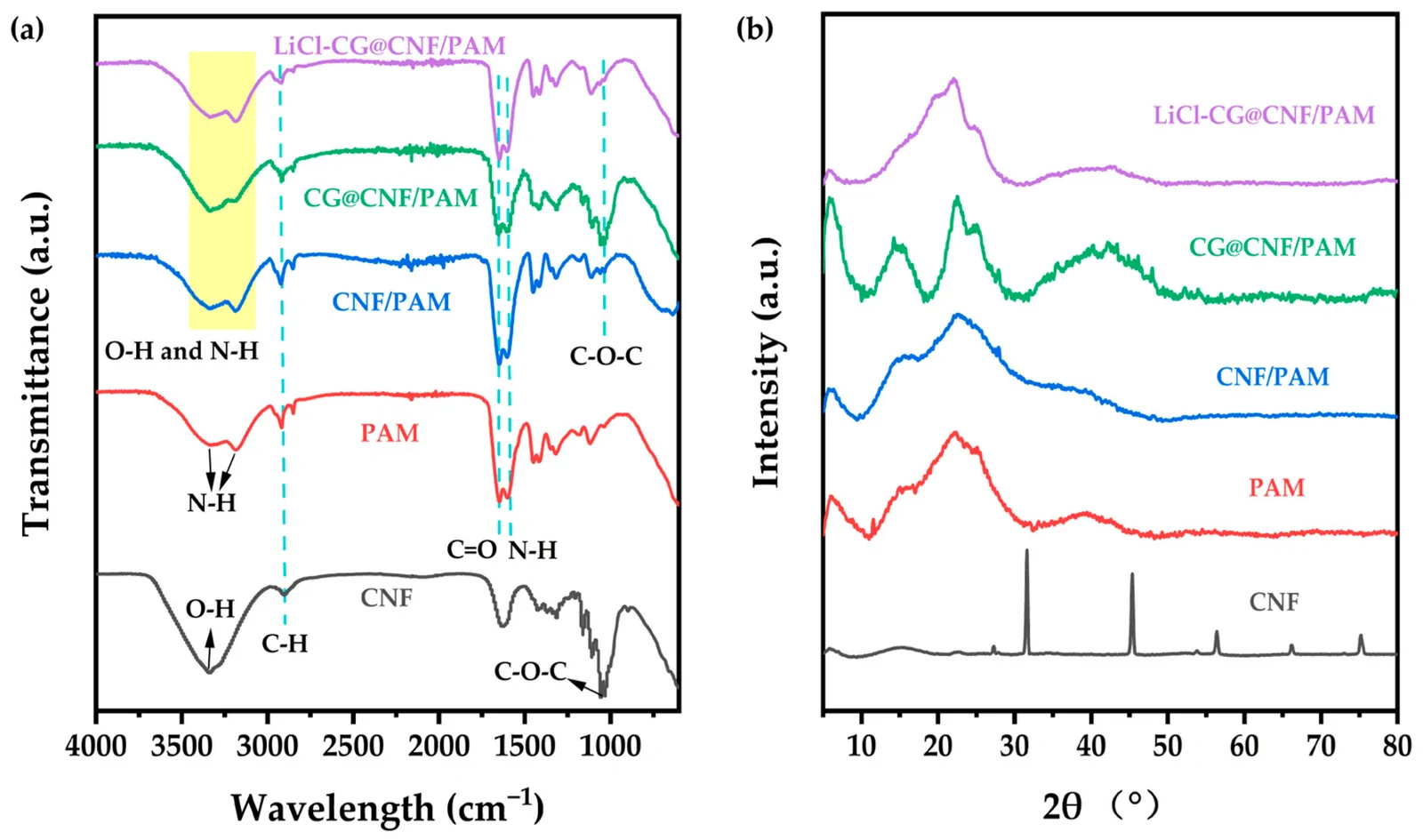
Characterization of hydrogels. (**a**) FTIR spectra of CNF, PAM, CNF/PAM, CG@CNF/PAM, LiCl-CG@CNF/PAM. (**b**) XRD patterns of CNF, PAM, CNF/PAM, CG@CNF/PAM, LiCl-CG@CNF/PAM.

Fourier transform infrared spectroscopy (FT-IR) was employed to analyze the chemical composition and intermolecular interactions in CNF, PAM, CNF/PAM, CG@CNF/PAM and LiCl-CG@CNF/PAM (Figure 2a). The stretching vibration peaks at 3338 cm^−1^ and 1160 cm^−1^ for CNF verify the existence of O-H groups and C-O-C glycosidic bonds. The PAM spectrum exhibits two characteristic N-H stretching peaks at 3187 cm^−1^ and 3338 cm^−1^ [27]. A C-H stretching vibration appears at 2924 cm^−1^, while the prominent bands at 1648 and 1604 cm^−1^ are respectively attributed to the C=O stretching vibration of the amide I band and t in-plane bending vibration of N-H in the amide II band [28]. Upon CNF incorporation, the peak at 1030 cm^−1^ for CNF/PAM, CG@CNF/PAM and LiCl-CG@CNF/PAM is assigned to the C-O-C glycosidic bond of CNF. The broad absorption band at 3073–3460 cm^−1^ arises from the overlap of O-H stretching vibrations of CNF and N-H stretching vibrations originating from PAM.

To further investigate the crystalline structures of CNF, PAM, CNF/PAM, CG@CNF/PAM, and LiCl-CG@CNF/PAM, X-ray diffraction (XRD) measurements were carried out (Figure 2b). CNF exhibits sharp, intense diffraction peaks at 2θ = 31.62°, 45.36°, and 56.28°, confirming its highly crystalline nature. A broad diffuse halo located at 22.26° is observed for PAM gel, which is characteristic of an amorphous polymer matrix. A broad diffuse halo assigned to amorphous PAM can be observed at 22.72° for CNF/PAM, 22.54° for CG@CNF/PAM, and 22.02° for LiCl-CG@CNF/PAM, demonstrating the preservation of the amorphous PAM matrix within these composite hydrogels. Due to the low CNF content in CNF/PAM, CG@CNF/PAM and LiCl-CG@CNF/PAM, no obvious characteristic diffraction peaks of CNF are observed in their XRD patterns.

**Figure 3:**
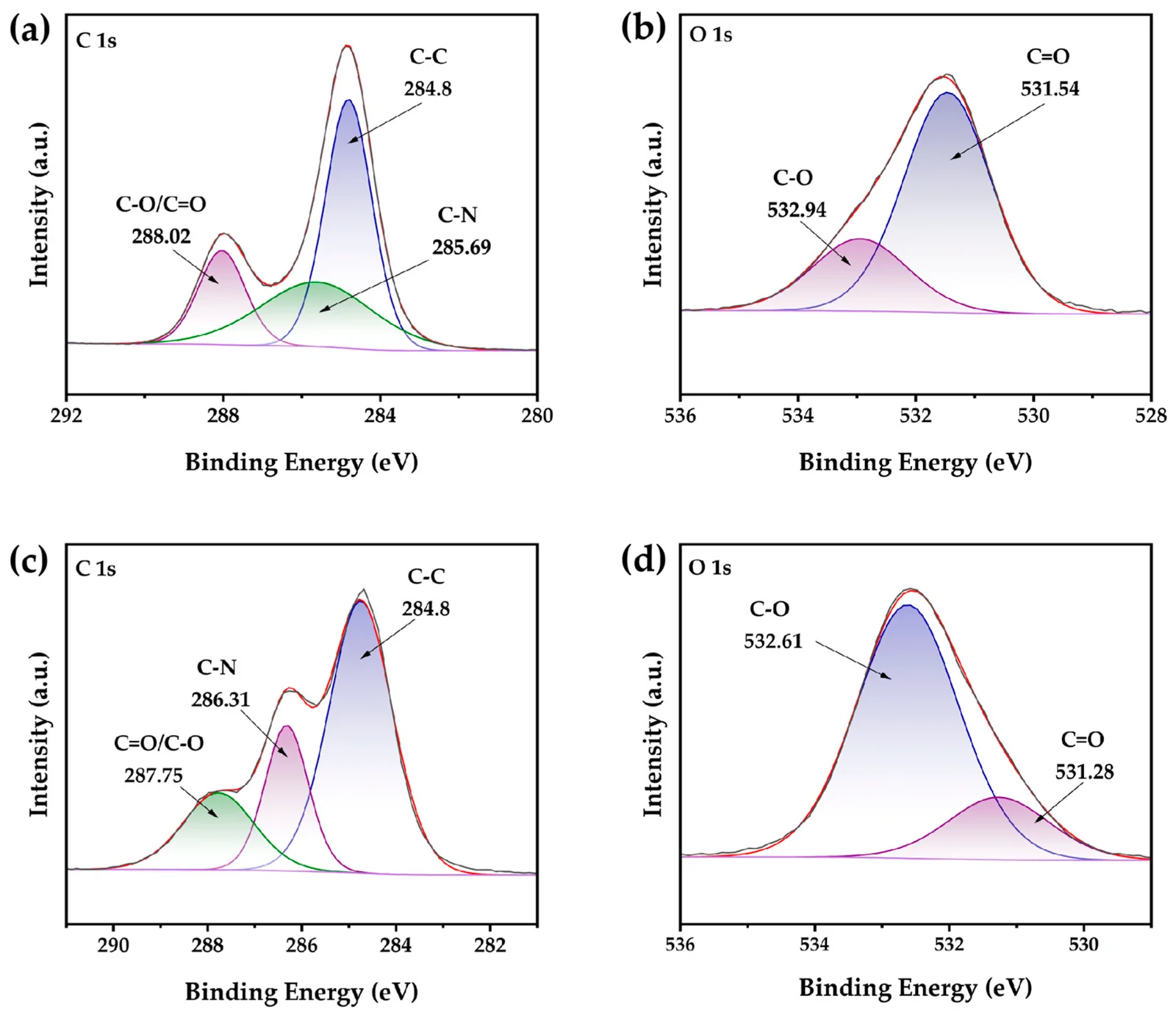
High-resolution XPS C 1s and O 1s spectra of CG@CNF/PAM and LiCl-CG@CNF/PAM hydrogels. (**a**) C 1s spectrum of CG@CNF/PAM. (**b**) O 1s spectrum of CG@CNF/PAM. (**c**) C 1s spectrum of -iCl-CG@CNF/PAM. (**d**) O 1s spectrum of LiCl-CG@CNF/PAM.

High-resolution XPS spectra reveal the chemical-state distributions of C 1s and O 1s in pristine CNF and four hydrogel systems. For CNF (Figure S1), the C 1s spectrum mainly consists of the C-C peak (284.8 eV) and the C-O peak (287.72 eV) originating from hydroxyl groups and glycosidic bonds. In the O 1s spectrum of CNF, the peak at 532.16 eV is attributed to C-O from hydroxyl and glycosidic bonds, and the peak at 536.04 eV is assigned to H-O-H from residual adsorbed water. The C 1s components of PAM hydrogel (Figure S2) include C-C (284.8 eV), C-N (285.31 eV) and C=O (288.15 eV) from amide bonds, whereas its O 1s spectrum is dominated by the C=O peak (531.69 eV). For the CNF/PAM hydrogel (Figure S3), the C 1s spectrum comprises C-C (284.8 eV), PAM-contributed C-N (285.25 eV), and the overlap between PAM-derived C=O and CNF-derived C-O at 288 eV. Its O 1s spectrum shows the C-O (532.01 eV) and C=O peak (531.38 eV). For the CG@CNF/PAM hydrogel (Figure 3a), the C 1s spectrum consists of C-C (284.8 eV), C-N from PAM (285.69 eV), C-O from CNF and C=O from PAM (288.02 eV). Its O 1s spectrum (Figure 3b) is composed of C-O (532.94 eV) and C=O (531.54 eV) originating from amide bonds and carboxyl groups. For the LiCl-CG@CNF/PAM hydrogel (Figure 3c), the C 1s spectrum contains the overlap (287.75 eV) between C-O and C=O, C-C (284.8 eV), and C-N(286.31 eV), while its O 1s spectrum (Figure 3d) comprises C-O (532.61 eV) and C=O (531.28 eV). Notably, the relative area fraction of C-O in the O 1s spectra of LiCl-CG@CNF/PAM increases to 80.37%, which is considerably higher than the 26.36% for CG@CNF/PAM. It is proposed that Li^+^ disrupts inter-fibril hydrogen bonding of cellulose via ion–dipole interactions, thereby exposing the intrinsically buried hydroxyl groups to the material surface [29].

**Figure 4:**
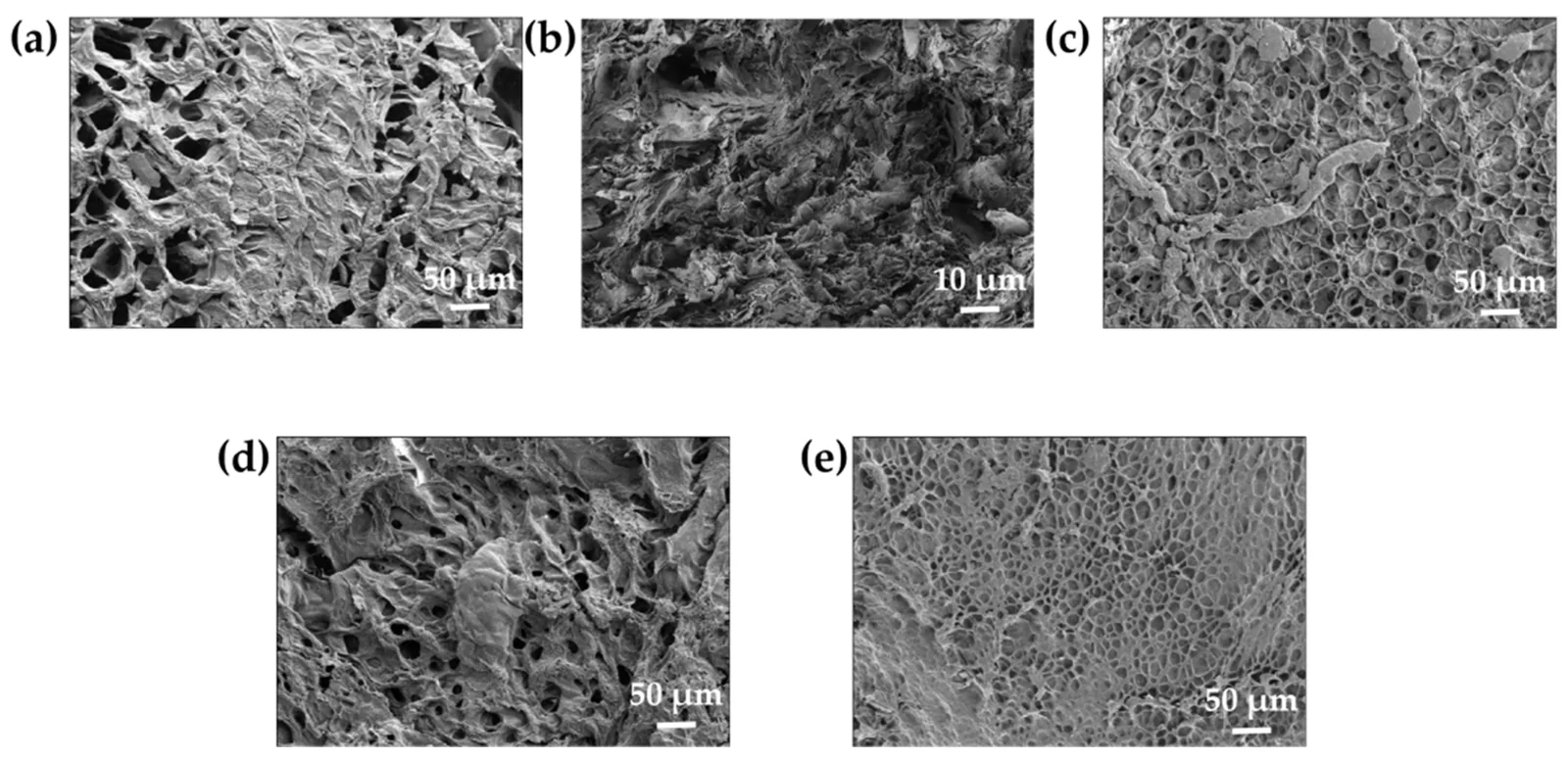
SEM images of CNF and hydrogels. SEM images of (**a**) PAM, (**b**) CNF, (**c**) CNF/PAM, (**d**) CG@CNF/PAM, and (**e**) LiCl-CG@CNF/PAM hydrogels.

Scanning electron microscopy (SEM) was performed on PAM, CNF/PAM, CG@CNF/PAM and LiCl-CG@CNF/PAM to investigate their microstructure. Pure PAM (Figure 4a) reveals large, heterogeneously distributed pores. CNFs (Figure 4b) display a highly entangled and interwoven ribbon-like fibrous network, where fibrils stack to form lamellar structures. CNF/PAM (Figure 4c) exhibits a refined honeycomb-like fibrous network with significantly reduced and more uniform pore sizes, indicative of a three-dimensional interpenetrating network stabilized by hydrogen bonding between CNF and PAM [30]. In CG@CNF/PAM (Figure 4d), graphene sheets are observed to be stacked along pore walls with localized CG aggregation, producing an uneven pore distribution. LiCl-CG@CNF/PAM (Figure 4e) presents the most uniform, high-density micropores, with markedly reduced aggregation-induced structural defects and continuous intact pore walls. This improved morphology suggests that the incorporation of Li^+^ enhances CG dispersion and promotes the formation of a highly interconnected hierarchical pore network.

### 3.2. Optimization of Various CNF Loadings

**Figure 5:**
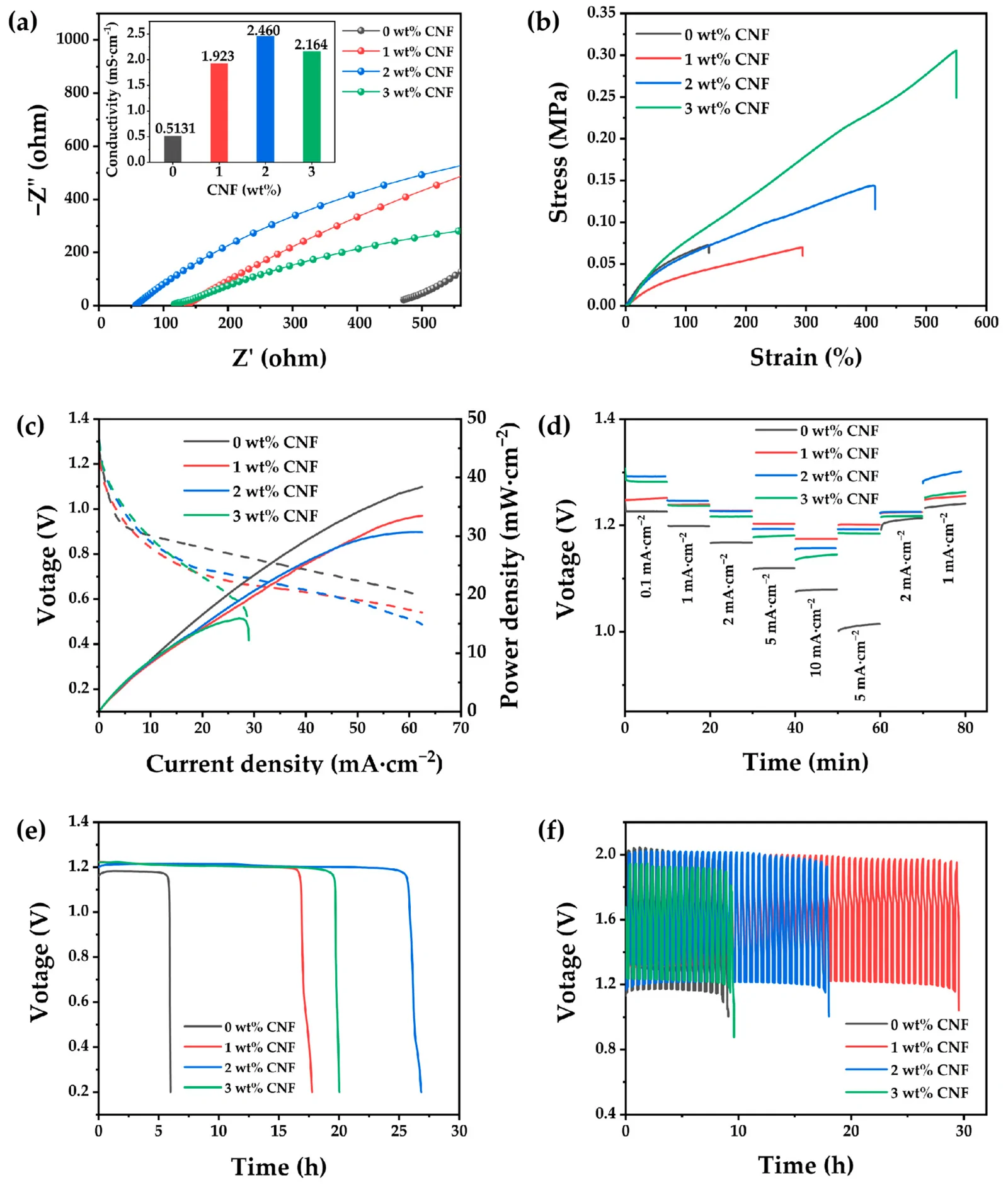
Characterization of PAM and CNF/PAM hydrogel electrolytes and the assembled ZABs. (**a**) AC impedance spectra and ionic conductivity values. (**b**) Tensile strength of the hydrogel electrolytes. (**c**) Discharge polarization profiles and corresponding power density curves. Solid lines represent discharge polarization profiles, and dashed lines represent corresponding power density curves. (**d**) Rate performance. (**e**) Galvanostatic discharge profiles. (**f**) GCD cycling curves at 2 mA·cm^−2^.

To enhance the structural stability and mechanical performance of PAM-based dual-network hydrogels, CNF was incorporated into PAM at 1, 2 and 3 wt% to construct a hydrogen-bonded network [31]. Ionic conductivity derived from AC impedance spectra is summarized in Figure 5a. The hydrogel containing 2 wt% CNF exhibited the highest ionic conductivity of 2.46 mS cm^−1^, whereas conductivity decreased at 3 wt%. With abundant surface hydroxyl groups and a high aspect ratio, cellulose nanofibrils (CNFs) facilitate the retention of substantial free water in the hydrogel while promoting the formation of interconnected honeycomb-like pores. These interconnected porous frameworks reduce the tortuosity of ion migration paths and facilitate sufficient electrolyte infiltration to reserve abundant mobile K^+^ and OH^−^, thus lowering the ion diffusion resistance inside the electrolyte matrix. The surface hydroxyls on CNFs interact with PAM chains and water to establish a three-dimensional hydrogen-bond network, suppressing water loss. In addition, the hydroxyl groups can form dynamic hydrogen bonds with PAM carbonyl and amide groups, creating a secondary physical crosslinking framework that supports rapid charge transfer without compromising the hydrogel’s structural integrity. However, excessive CNF loading can lead to nanofiber agglomeration, pore blockage and increased gel viscosity, ultimately detracting from the ionic conduction performance of the composite electrolyte.

Tensile tests of CNF/PAM hydrogels (Figure 5b) were implemented to validate the reinforcing function of CNF nanofibers. The statistical results of tensile strength and elongation at break with error bars (mean ± SD, *n* = 3) are provided in Figure S4 to demonstrate the reproducibility of mechanical measurements. The results show that the 3 wt% CNF hydrogel achieves the highest toughness with a fracture stress of 305.4 kPa and a fracture strain of 549%. Such prominent mechanical performance originates from the high-modulus CNF skeleton, which enables stepwise hydrogen bond dissociation to consume external mechanical energy [32]. Uniformly dispersed CNFs bear a substantial portion of the applied stress and redistribute load within the PAM matrix under tensile loading, thereby mitigating local stress concentrations. The dynamic hydrogen bonds formed among CNF surface hydroxyls, PAM amide groups and ambient water undergo progressive dissociation rather than abrupt cleavage under strain. This graded bond-breaking continuously dissipates external mechanical energy, suppresses crack propagation, and delays irreversible damage to the gel framework. Collectively, this staged energy-absorbing behavior markedly enhances the stretchability and fracture resistance of the dual-network hydrogel system.

Figure 5c compares the galvanic polarization of ZABs based on PAM and CNF-reinforced PAM electrolytes. Cells incorporating CNF/PAM hydrogel exhibit larger discharge voltage gaps and inferior power density compared with the PAM-based batteries. This deterioration is primarily attributable to increased gel viscosity following CNF addition, which impedes O_2_ transport to the catalytic air cathode. The oxygen reduction reaction suffers severe mass-transport limitations, which intensify concentration polarization of K^+^ and OH^−^ and broadens the voltage gap between charge and discharge.

Electrochemical tests, including rate capability, specific capacity and long-term cycling, were conducted at 2 mA·cm^−2^. Figure 5d records that varying CNF content induces only minor changes in rate performance, with both 1 wt% and 2 wt% CNF composites sustaining high and stable discharge plateaus. CNF_2wt%_/PAM-based ZABs achieve a longer discharge time of 26 h (Figure 5e), while CNF_1wt%_/PAM-based cells demonstrate the best cycling performance, sustaining 58 cycles (29 h) at 2 mA·cm^−2^ (Figure 5f). Overall, dual-network CNF/PAM hydrogels outperform single-component PAM in most electrochemical metrics. These improvements arise from two complementary structural effects of CNFs: (1) the interwoven hydrogen-bonded network shortens ion migration pathways, accelerates charge-carrier transport and mitigates ohmic and concentration polarization, thereby enhancing capacity and rate capability; and (2) a rigid, high-modulus CNF scaffold mechanically reinforces the gel and preserves intimate electrode–electrolyte contact during cycling, suppressing uneven zinc deposition and dendrite growth and extending device lifetime.

### 3.3. Gradient CG Modification on CNF-Reinforced Hydrogel Matrices

CG was incorporated into the above hydrogel systems to explore the mechanical and electrochemical performances of the ternary composite hydrogel electrolytes. As revealed in the above investigations, 2 wt% CNF yields optimal specific capacity, whereas 1 wt% CNF affords the longest cycle life. To investigate the compatibility of hydrogels with varying CNF loadings within the ternary composite modification system, gradient doping of CG was performed on hydrogel substrates containing 1 wt% and 2 wt% CNFs, synthesizing a set of CG@CNF/PAM hydrogel electrolytes and characterizing the assembled ZABs to screen out the optimal component ratio.

The electrochemical impedance spectroscopy measurements for the hydrogels were conducted in a sandwich-structured cell comprising dual copper electrodes (Cu/gel/Cu). The zinc–air battery configuration employed is illustrated in Figure 4a: zinc foil served as the anode, while the cathode was a nickel foam current collector loaded with Co_3_O_4_ catalyst. The hydrogel electrolyte was positioned between the anode and cathode, and the entire assembly was sealed with a porous plastic separator. Then the performances of CG@CNF_1wt%_/PAM and CG@CNF_2wt%_/PAM hydrogel electrolytes were measured and compared.

When CNF was fixed at 1 wt%, CG addition (Figures S5 and S6) significantly increased ionic conductivity and mechanical toughness relative to CNF_1wt%_/PAM. However, power density, rate capability, specific capacity and cycle life of the corresponding ZABs all declined. The insufficient 1 wt% CNF fiber scaffold fails to provide adequate network support for CG dispersion, leading to localized CG aggregation that disrupts the original pore structure and interfacial stability, preventing synergistic conductivity enhancement and structural optimization. Thus, 2 wt% CNF was selected as the baseline formulation for subsequent multi-component studies.

**Figure 6:**
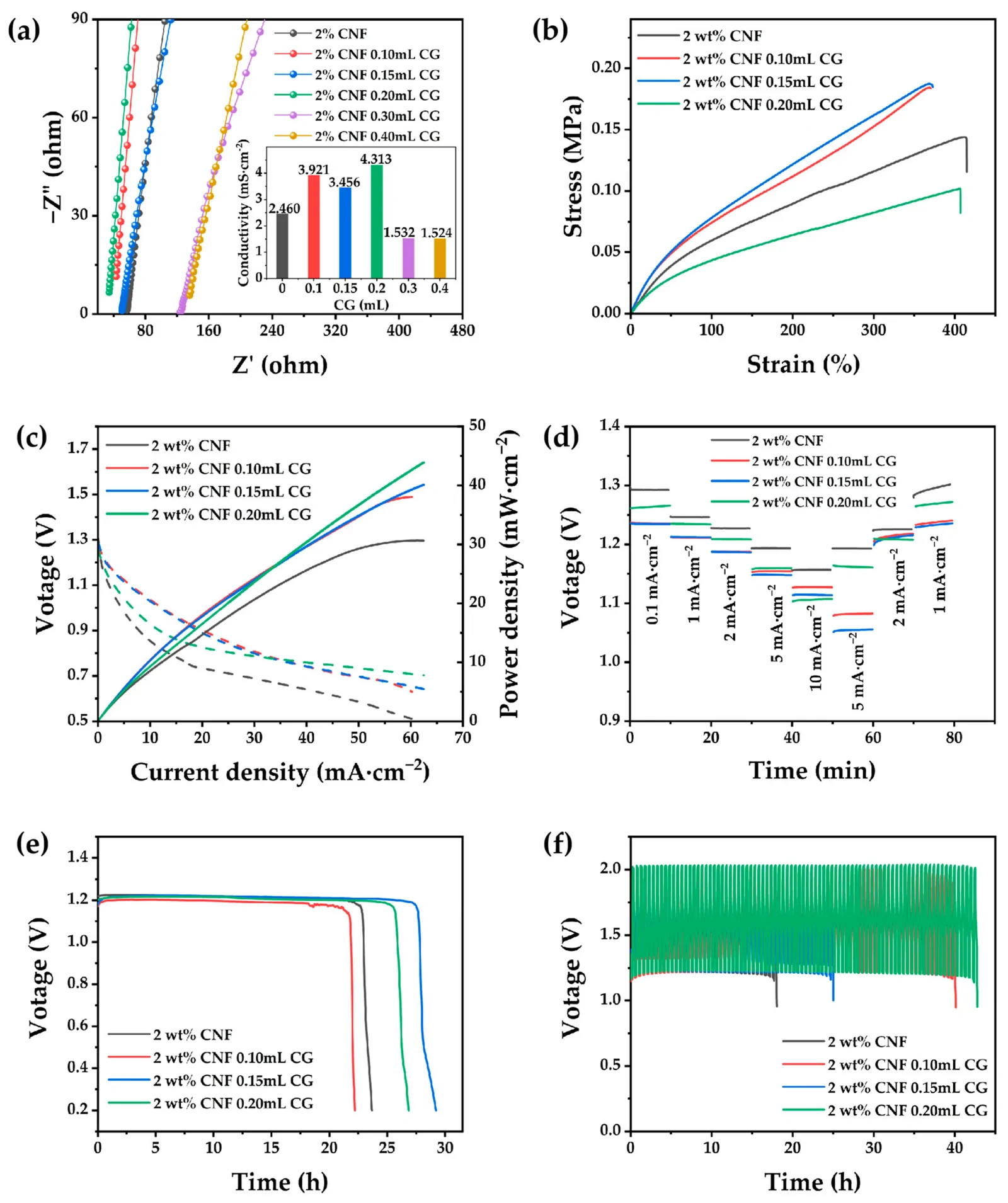
Characterization of CNF_2wt%_/PAM and three CG@CNF_2wt%_/PAM hydrogel electrolytes and the assembled ZABs. (**a**) AC impedance spectra and ionic conductivity values. (**b**) Tensile strength of the hydrogel electrolytes. (**c**) Discharge polarization profiles and corresponding power density curves. Solid lines represent discharge polarization profiles, and dashed lines represent corresponding power density curves. (**d**) Rate performance. (**e**) Galvanostatic discharge profiles. (**f**) GCD cycling curves at 2 mA·cm^−2^.

With CNF content fixed at 2 wt%, the ionic conductivity of the composite hydrogel with CG additions of 0.10 mL, 0.15 mL, 0.20 mL, 0.30 mL, and 0.40 mL was measured by recording the EIS of AC impedance spectra (Figure 6a). These results indicate that a CG content of 0.20 mL yields the highest ionic conductivity, reaching 4.313 mS·cm^−1^. Incorporating CG into the CNF/PAM dual-network matrix transforms the hydrogel from a single ionic conductor to a synergistic ion-electron co-conductive system. Notably, once the CG loading surpasses the percolation threshold, the planar π-conjugated CG nanosheets readily interlock with each other to construct a continuous three-dimensional electron-transport network, and electron tunneling between adjacent nanosheets provides extra conductive pathways. However, CG aggregation becomes pronounced when CG content exceeds 0.20 mL, leading to extensive particle agglomeration that blocks a large fraction of pores and sharply increases ionic transport resistance.

The three-component composite hydrogels with CG additions of 0.10 mL and 0.15 mL exhibit higher fracture strength than the CNF_2wt%_/PAM hydrogel (Figure 6b and Figure S7). CG_0.15_@CNF_2wt%_/PAM exhibited the best tensile performance with a fracture stress of 187 kPa at a strain of 368%. Abundant intermolecular hydrogen bonding is formed between carboxyl groups of CG and hydroxyl groups on CNF, constructing a dense physically crosslinked network. In addition, incorporating rigid CG nanosheets strengthens the CNF skeleton, promoting effective stress transfer and dissipation across the hydrogel network, thereby markedly increasing its fracture strength. However, when the CG dosage reaches 0.20 mL, excessive CG nanosheets disrupt the originally continuous and ordered CNF load-bearing network, generating numerous interfacial defects and stress concentration points, which lead to a sharp drop in tensile strength.

The power density, rate capability, specific capacity and cycling lifespan of the assembled ZABs were evaluated. As shown in Figure 6c, discharge polarization curves and the corresponding power density curves for three CG-modified hydrogel electrolytes were characterized. The ZAB assembled with CG_0.2_@CNF_2wt%_/PAM delivered the smallest discharge voltage gap and the maximum power density of 43.9 mW·cm^−2^. CG nanosheets and CNFs form conductive networks and ion-transport channels, optimizing charge transfer and reducing internal resistance and polarization. This enables a stable discharge voltage and low energy loss for the system. The batteries were discharged at various current densities, with results shown in Figure 6d. The hydrogel electrolyte without CG exhibited a higher discharge plateau and greater stability. This behavior arises because two-dimensional CG sheets tend to agglomerate and stack, blocking the ion-transport pores inside the hydrogel. Moreover, CG agglomeration increases interfacial impedance and reduces the water-retention capacity, leading to severe polarization under high-rate discharge and a diminished discharge plateau and stability. Galvanostatic discharge profiles of ZABs based on CG@CNF_2wt%_/PAM hydrogels are presented in Figure 6e. The battery with 0.15 mL CG achieved the highest discharge plateau of 1.21 V and the longest discharge duration of 29 h. Galvanostatic charge–discharge curves tested at a current density of 2 mA·cm^−2^ (Figure 6f) show that the ZAB employing CG_0.2_@CNF_2wt%_/PAM possesses outstanding rechargeability and superior cycling stability, sustaining 84 cycles (42 h). Overall, ZABs assembled with CG-containing hydrogel electrolytes exhibited better cycling performance than those using CG-free hydrogel electrolytes. This improvement can be explained by the multifunctional effects of CG: (1) CG establishes an efficient conductive network to accelerate electron transfer and expand reactive interfaces, improving active electrode-site utilization and thus boosting battery-specific capacity; (2) the surface carboxyl groups of CG exert an electrostatic shielding effect to homogenize Zn^2+^ deposition and suppress zinc dendrite proliferation; the negatively charged carboxyl sites also regulate the migration rate of surrounding K^+^ and balance OH^−^ flux on the zinc anode surface. Its two-dimensional sheet structure stabilizes the electrode–electrolyte interface, and hydrogen bonding retains water to slow electrolyte dehydration. These synergistic effects suppress parasitic electrode reactions and substantially enhance cycling stability. In summary, the CG_0.2_@CNF_2wt%_/PAM hydrogel serves as the optimal electrolyte among all ternary composite hydrogel systems investigated in this work.

### 3.4. Synergistic LiCl Doping Mediating Ionic Transport and Mechanical Toughness

**Figure 7:**
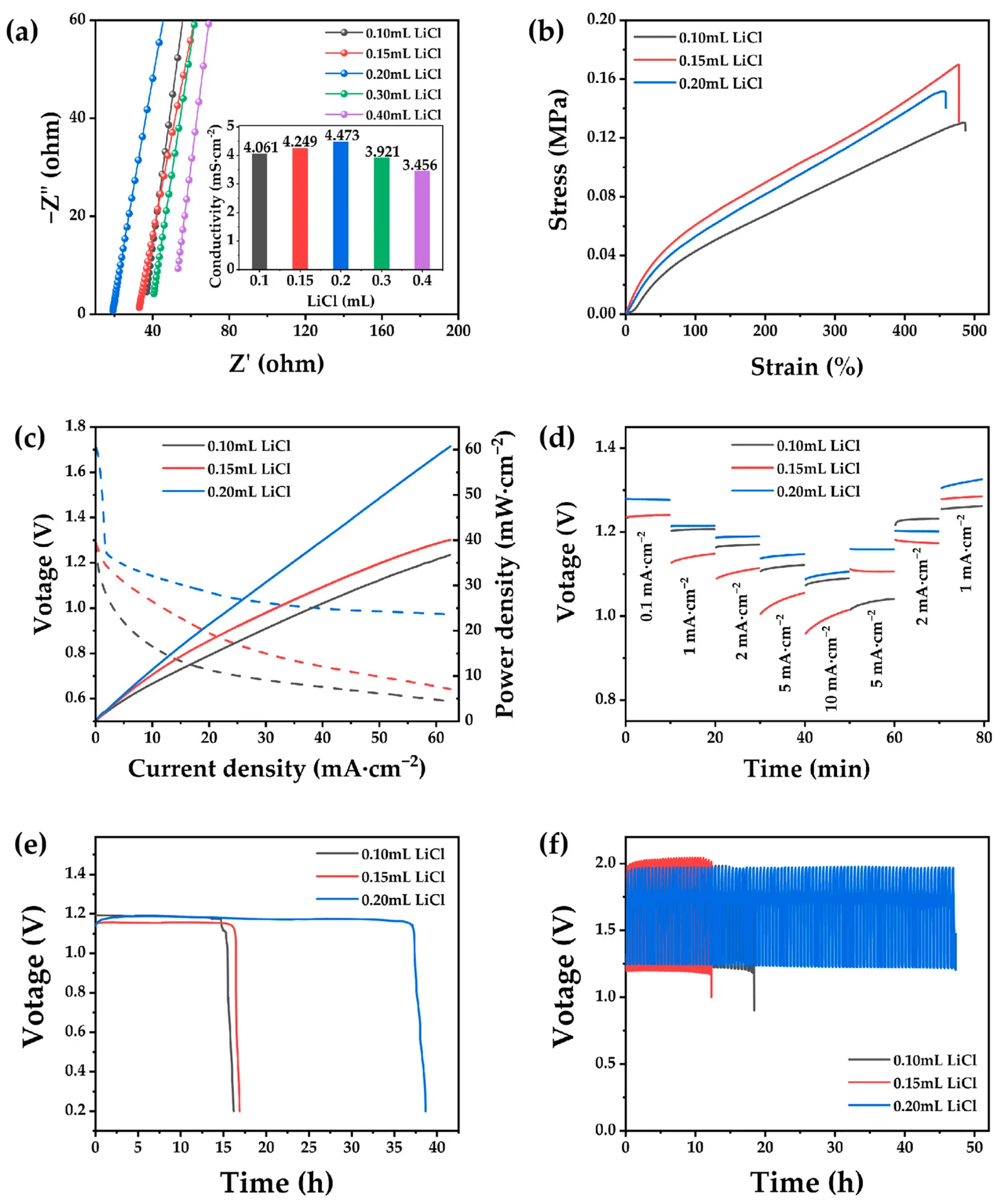
Characterization of three LiCl-CG_0.2_@CNF_2wt%_/PAM hydrogel electrolytes and the assembled ZABs. (**a**) AC impedance spectra and ionic conductivity values. (**b**) Tensile strength of the hydrogel electrolytes. (**c**) Discharge polarization profiles and corresponding power density curves. Solid lines represent discharge polarization profiles, and dashed lines represent corresponding power density curves. (**d**) Rate performance. (**e**) Galvanostatic discharge profiles. (**f**) GCD cycling curves at 2 mA·cm^−2^.

To further improve ionic transport within the hydrogel electrolyte, LiCl was incorporated into the CG_0.2_@CNF_2wt%_/PAM network to construct LiCl-CG_0.2_@CNF_2wt%_/PAM multi-component composite hydrogel electrolytes. AC impedance spectra were collected for hydrogels containing different LiCl additions of 0.10, 0.15, 0.20, 0.30, and 0.40 mL (Figure 7a). LiCl_0.2_-CG_0.2_@CNF_2wt%_/PAM exhibits the highest ionic conductivity of 4.473 mS· cm^−1^, slightly higher than that of the LiCl-free CG_0.2_@CNF_2wt%_/PAM hydrogel. In contrast, both lower and higher LiCl dosages lead to reduced conductivity, and a pronounced conductivity decrease is observed when the LiCl dosage reaches 0.30 mL or above. This trend indicates that LiCl does not simply enhance conductivity by increasing salt concentration; instead, its effect is governed by the competition between the generation of additional mobile ions and the structural restriction imposed on the hydrogel network. Abundant K^+^ and OH^−^ tend to form ionic clusters via electrostatic association. At the optimal LiCl dosage of 0.20 mL, dissociated Li^+^ with high charge density uniformly distributes within gel pores. These Li^+^ ions separate K^+^ from OH^−^ through electrostatic repulsion, restrain their aggregation and ion pairing, raise the proportion of free K^+^ and OH^−^ in the system, and augment the quantity of mobile charge carriers available for ion transport. Uniformly dispersed Li^+^ tailor the hierarchical porous network to mitigate pore blockages originating from carboxylated graphene agglomeration and construct low-resistance transport channels for K^+^ and OH^−^. The dissociated free Li^+^ ions, characterized by their high charge density, encounter a milieu rich in electronegative polar functional groups, including amide moieties along the polyacrylamide backbone, hydroxyl groups on the cellulose nanofiber surfaces, and carboxyl groups borne by the carboxylated graphene nanosheets. Drawing on extensive relevant literature [33,34,35], we tentatively propose that Li^+^ may engage with these polar groups to form reversible ion–dipole non-covalent interactions. Driven by this ion–dipole coordination, the hydrated Li^+^ species are postulated to modulate the local hydration microenvironment within the gel matrix and facilitate the assembly of interconnected hydrated ion-transport channels, thereby accelerating ion diffusion kinetics. In this case, Li^+^ and Cl^−^ migrate through interconnected aqueous channels, while polar groups on PAM, CNF and CG provide coordination and solvation sites that assist ion transport. As a result, the electrolyte resistance is reduced, and the macroscopic ionic conductivity is improved.

Tensile tests (Figure 7b) confirm the reinforcing effect of LiCl, and the corresponding statistical data (error bars for tensile strength and elongation at break, *n* = 3) in Figure S8 demonstrate that hydrogels incorporated with LiCl exhibit higher tensile strength than LiCl-free CG_0.2_@CNF_2wt%_/PAM. The maximum tensile strength is obtained at a LiCl dosage of 0.15 mL, with a fracture stress of 169 kPa at a fracture strain of 477%. Li^+^ cations are inferred to form reversible ion–dipole noncovalent coordination bonds with amide, hydroxyl and carboxyl groups, introducing additional physical crosslinking sites alongside the inherent hydrogen-bond network. The reversible dissociation and reformation of these ion–dipole interactions dissipate external energy during stretching and thus enhance the toughness of the hydrogel.

Power density curves (Figure 7c) show that LiCl-CG_0.2_@CNF_2wt%_/PAM achieves a maximum power density of 60.7 mW·cm^−2^, significantly higher than the 43.9 mW·cm^−2^ for CG_0.2_@CNF_2wt%_/PAM. Rate-performance curves at different current densities (0.1, 1, 2, 5, 10, 5, 2 mA cm^−2^) (Figure 7d) demonstrate that LiCl-CG_0.2_@CNF_2wt%_/PAM delivers higher and more stable discharge plateaus. Galvanostatic discharge curves at 2 mA·cm^−2^ (Figure 7e) reveal that LiCl_0.2_-CG_0.2_@CNF_2wt%_/PAM-based ZABs achieve the highest specific capacity, with a 38 h discharge time, representing a 31.0% improvement over the 29 h observed for CG_0.2_@CNF_2wt%_/PAM-based cells, confirming the superior efficiency of the LiCl_0.2_-CG_0.2_@CNF_2wt%_/PAM electrolyte. GCD curves (Figure 7f) indicate that this electrolyte sustains stable cycling for approximately 47 h with a voltage gap of 0.76 V, demonstrating excellent cycling stability and voltage-output consistency, surpassing CG_0.2_@CNF_2wt%_/PAM and confirming that LiCl enhances cycling durability.

The overall electrochemical performance of the battery is markedly enhanced by the synergistic optimization of ionic transport and microstructure in the hydrogel electrolyte achieved through LiCl doping. Moderately introduced Li^+^ ions, owing to their small size and high mobility, increase the concentration of mobile charge carriers, reorganize hydrated conduction pathways, and streamline ion migration routes, thereby significantly reducing the ohmic resistance of the electrolyte. The optimized conductive network effectively mitigates ohmic and concentration polarization under high-amplitude currents and high-rate operation, boosting peak power density and adaptability to demanding current conditions. Moreover, interconnected porous structures deliver active ions to the electrode interface during prolonged discharge, promoting rapid redox reactions at the electrode surface, extending discharge duration, and enhancing practical capacity utilization. From a structural and interfacial perspective, Li^+^ ions introduced by doping are proposed to establish ion–dipole coordination interactions with polar functional groups (amide, hydroxyl, and carboxyl) within the composite hydrogel. These reversible noncovalent coordination bonds serve as energy-consuming sacrificial sites under cyclic tensile strain during repeated charging and discharging. They dissipate mechanical stress imposed on the gel framework, which enhances the composite’s intrinsic toughness and cyclic structural durability. Mechanically strengthened gel substrates maintain tight adhesion against electrode surfaces throughout prolonged cycling. This prevents interfacial separation and the corresponding surge of contact resistance induced by continuous structural distortion. A well-stabilized solid–liquid boundary homogenizes current flux across the zinc anode surface, erases localized current hot zones, moderates irregular metal sedimentation, restrains dendrite propagation, and lowers the risks of progressive interfacial decay and internal short circuits. In addition, the finely tuned crosslinking network restricts water evaporation within the hydrogel, effectively alleviating electrolyte drying and structural deterioration. The synergistic advancement of ionic migration kinetics, mechanical robustness, and electrode interfacial stability collectively endows the LiCl-modified electrolyte with superior power output, rate adaptability, sustained discharge performance and long-cycle durability.

To clearly elucidate the comprehensive optimization strategy for the hydrogel electrolyte, the key performance metrics of various formulations, including ionic conductivity, tensile properties, peak power density, and cycling stability, are summarized in Table 1. The LiCl_0.2_-CG_0.2_@CNF_2wt%_/PAM composition is ultimately selected, as it achieves an optimal balance between mechanical strength and electrochemical performance through synergistic multi-component interactions. First, the incorporation of 2 wt% CNF establishes a foundational mechanical network that reinforces the gel matrix and refines the porous architecture. Subsequently, the addition of 0.20 mL CG markedly enhances the ionic conductivity (from 2.460 to 4.313 mS·cm^−1^) and cycling stability (from 29 h to 42 h), yet at the cost of partial deterioration in mechanical performance. Finally, the introduction of 0.20 mL LiCl not only marginally elevates the ionic conductivity to 4.473 mS·cm^−1^, but more importantly, it synergistically restores the tensile stress from 101 kPa back to 151.5 kPa, while substantially boosting both the peak power density (from 43.9 to 60.7 mW·cm^−2^) and the galvanostatic discharge duration (from 26 to 38 h). The combined effects of these three optimal dosages yield the best overall performance among all formulations investigated.

A comprehensive comparison of the developed LiCl-CG@CNF/PAM hydrogel with various reported hydrogel electrolyte systems is summarized in Table 2. Most reported studies are restricted to pure cellulose, cellulose–graphene or cellulose–LiCl binary systems, and the synergistic modification with ternary components of cellulose nanofibrils, graphene-based filler, and LiCl has been rarely explored. Although this study successfully balances superior mechanical strength with competitive electrochemical output, it admittedly exhibits relatively low ionic conductivity and insufficient long-term cycling stability, which remain critical challenges to be addressed in future modifications.

## 4. Conclusions

A multi-component dual-crosslinked hydrogel, LiCl-CG@CNF/PAM, was fabricated via a rationally designed interpenetrating network, consisting of a covalently crosslinked PAM backbone intertwined with a dynamic secondary network stabilized by hydrogen bonds between CNF and CG, together with reversible Li^+^–dipole interactions. This hierarchical architecture simultaneously endows the hydrogel with robust mechanical integrity, rapid ion transport, and durable electrode–electrolyte interfacial stability. CNF creates a uniform, interconnected honeycomb-like porosity that reinforces the matrix and shortens ion migration pathways. CG enables an ion-electron transport network while mitigating zinc dendrite growth through electrostatic shielding, thereby improving cycling stability. Li^+^ raises the concentration of mobile charge carriers and are deduced to form ion–dipole interactions with polar amide and hydroxyl groups, forming reversible crosslinks that dissipate mechanical stress and stabilize the solid–liquid interface. The ion–dipole forces induced by Li^+^ also enhance CG nanosheets dispersion, suppress aggregation, and promote a continuous hierarchical porous structure. The optimized composition LiCl_0.2_-CG_0.2_@CNF_2wt%_/PAM yields an ionic conductivity of 4.473 mScm^−1^ and notable stretchability. In flexible Zn–air batteries, the hydrogel delivers a peak power density of 60.7 mW cm^−2^, a discharge duration of 38 h, and a cycling lifetime of 47 h. This work outlines a generalizable multiscale strategy for designing mechanically robust and ionically efficient hydrogel electrolytes to guide next-generation flexible energy storage systems.

## Figures and Tables

**Table 1:** Comparative summary of key performance parameters of gradient-modified hydrogel electrolytes for optimal formulation screening.

Sample	Conductivity (mS·cm^−1^)	Stress/Elongation	Power Density (mW·cm^−2^)	Galvanostatic Discharge Duration (h)	Cycling Stability (h)
PAM	0.5131	72 kPa/138%	38.4	6	9.6
CNF_1wt%_/PAM	1.923	62 kPa/296%	33.5	17	29
CNF_2wt%_/PAM	2.460	143 kPa/414%	30.6	26	18
CNF_3wt%_/PAM	2.164	305 kPa/549%	15.9	20	9
CG_0.1_@CNF_1wt%_/PAM	2.291	96 kPa/358%	31.5	5	21
CG_0.15_@CNF_1wt%_/PAM	2.321	113 kPa/440%	29.4	11	8.6
CG_0.2_@CNF_1wt%_/PAM	2.125	110 kPa/393 wt%	25.4	6	11.5
CG_0.1_@CNF_2wt%_/PAM	3.921	184 kPa/369%	38.1	22	40
CG_0.15_@CNF_2wt%_/PAM	3.456	187 kPa/368%	40.1	29	25
CG_0.2_@CNF_2wt%_/PAM	4.313	101 kPa/407%	43.9	26	42
LiCl_0.1_-CG_0.2_@CNF_2wt%_/PAM	4.061	130 kPa/485%	36.8	15	18
LiCl_0.15_-CG_0.2_@CNF_2wt%_/PAM	4.249	169 kPa/477%	40.2	16	12
LiCl_0.2_-CG_0.2_@CNF_2wt%_/PAM	4.473	151 kPa/456%	60.7	38	47

**Table 2:** Comparison of comprehensive performance of reported hydrogel electrolytes for flexible zinc–air batteries.

Electrolyte System	Ionic Conductivity (mS·cm^−1^)	Fracture Stress/Elongation	Power Density (mW·cm^−2^)	Cycling Stability (h)	Ref.
LiCl-CG@CNF/PAM (this work)	4.473	151.50 kPa/457%	60.7	47	this work
PAM/CNF	391.24	≈35 kPa/2900%	34.7	Not reported	[36]
PAM/CMC/CNF	290	54.64 kPa/250%	82.58	30	[37]
PANa-cellulose	280	>500 kPa/>1000%	210.5	110	[38]
PAM/CMC/GO	306	68.1 kPa/350%	68.7	24	[19]
PAM/CNF/LiCl	997	90 kPa/748%	≈1.5	10,000 cycles	[39]
CNF/FHE (PANa/CNF/LiCl)	330.1	20–25 kPa/250–300%	30	80	[40]

## Data Availability

The original contributions presented in this study are included in the article/Supplementary Materials. Further inquiries can be directed to the corresponding author.

## Supplementary Materials

The following supporting information can be downloaded at: https://www.mdpi.com/article/10.3390/polym18182243/s1, Figure S1. High-resolution XPS C 1s and O 1s spectra of CNF. Figure S2. High-resolution XPS C 1s and O 1s spectra of PAM hydrogel. Figure S3. High-resolution XPS C 1s and O 1s spectra of CNF/PAM hydrogel. Figure S4. Tensile strain and strength of PAM and CNF/PAM hydrogel. Figure S5. Characterization of CNF_1wt%_/PAM and CG@CNF_1wt%_/PAM hydrogel electrolytes and the assembled ZABs. Figure S6. Tensile strain and strength of CNF_1wt%_/PAM and CG@CNF_1wt%_/PAM hydrogel. Figure S7. Tensile strain and strength of CNF_2wt%_/PAM and CG@CNF_2wt%_/PAM hydrogel. Figure S8. Tensile strain and strength of LiCl-CG_0.2_@CNF_2wt%_/PAM hydrogel.
