# Supplementary material for: Synergistically Modified CNF/PAM Hydrogel with LiCl and Carboxylated Graphene for Zinc–Air Batteries
Source: Polymers (Basel). 2026 Sep 15;18(18):2243. doi: 10.3390/polym18182243 (PMC13611287; doi:10.3390/polym18182243)
Supplement: Supplementary file 1 [file polymers-18-02243-s001.zip › polymers-4449866-supplementary.pdf]

Supplementary Materials

Synergistically Modified CNF/PAM Hydrogel with LiCl and Carboxylated Graphene for Zinc–Air Batteries

Weiyue Liu [1,2], Mingxuan Liu[1,2], Luqing Zhang[1,2], Sitong Guo[1,2], Yifeng Ruan[1,2], Ruiwen Wang[1,2], Chenhui Yang [1,2,*]

1 State Key Laboratory of Utilization of Woody Oil Resource, Northeast Forestry University, Harbin 150040, China; 2023220496@nefu.edu.cn (W.L.); liumx@nefu.edu.cn (M.L.); 17535714662@163.com (L.Z.); gst826293283@163.com (S.G.); 18745447299@163.com (Y.R.); wrwnefu@163.com (R.W.)
2 Key Laboratory of Bio-Based Material Science & Technology of Ministry of Education, Northeast Forestry University, Harbin 150040, China
*Correspondence: yangch@nefu.edu.cn

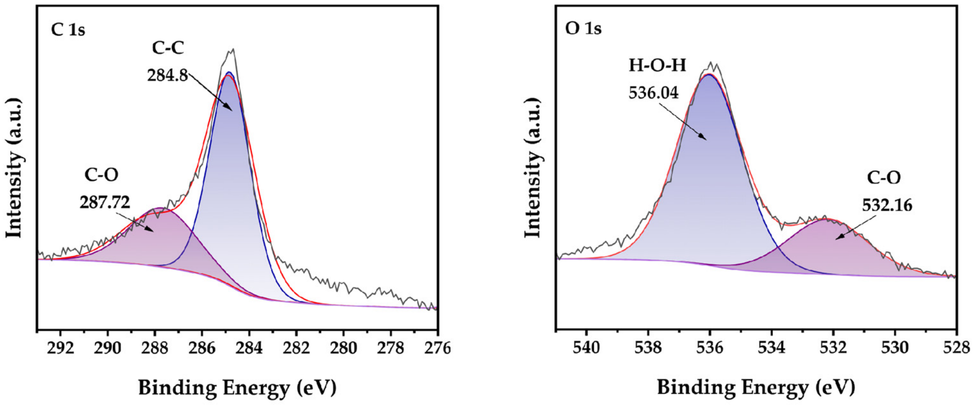

Figure S1. High-resolution XPS C 1s and O 1s spectra of CNF.

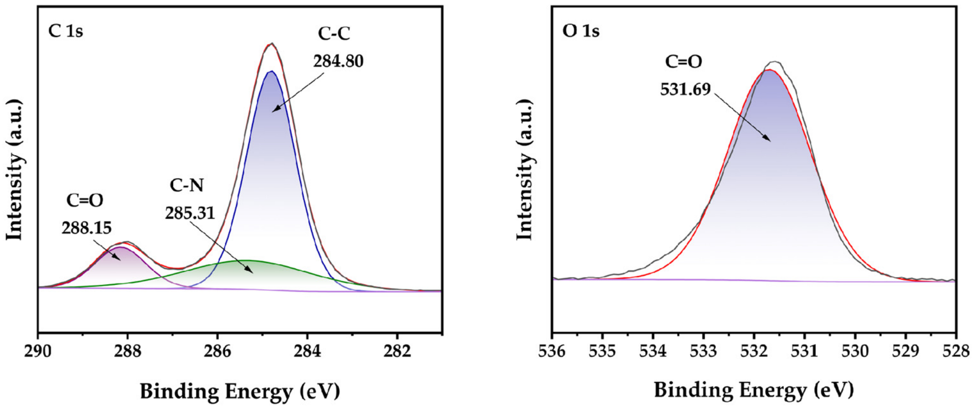

Figure S2. High-resolution XPS C 1s and O 1s spectra of PAM hydrogel.

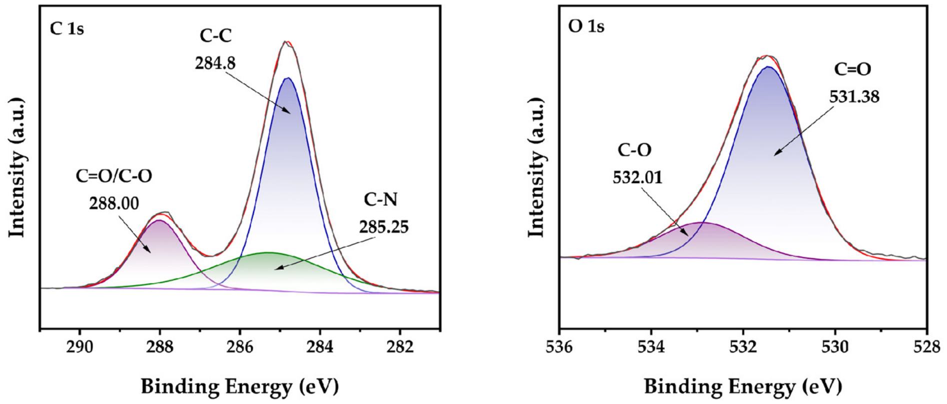

Figure S3. High-resolution XPS C 1s and O 1s spectra of CNF/PAM hydrogel.

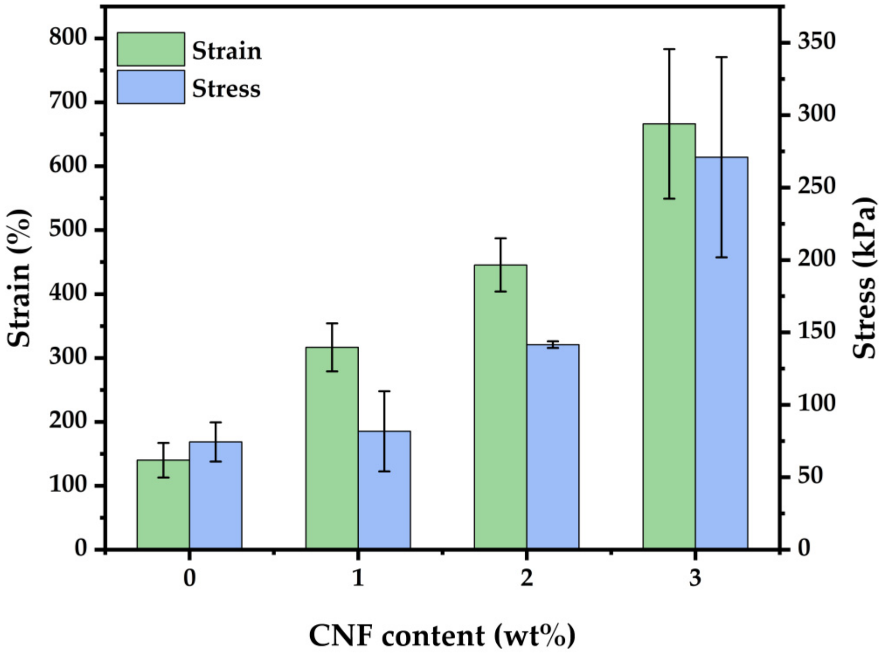

Figure S4. Tensile strain and strength of PAM and CNF/PAM hydrogel. Data with error bars are presented as mean values ± standard deviations of n = 3 replicates.

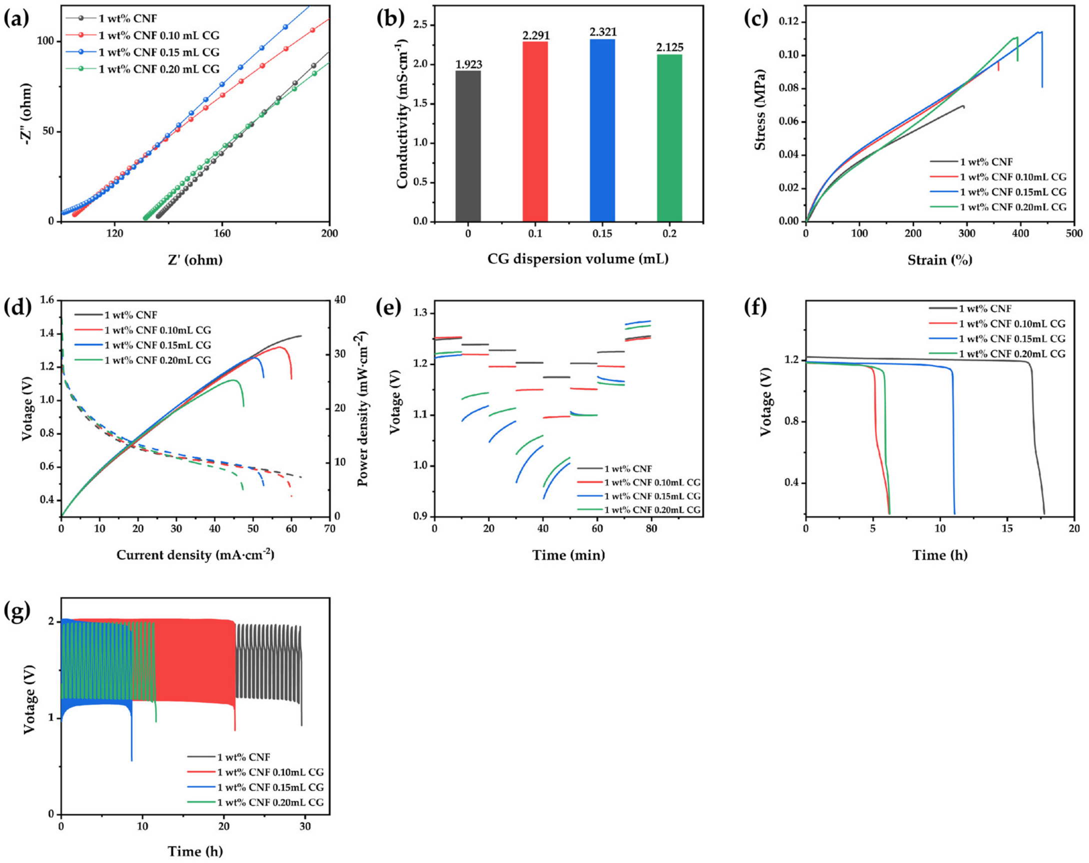

Figure S5. Characterization of CNF$_{1\ wt\%}$/PAM and CG@CNF$_{1\ wt\%}$/PAM hydrogel electrolytes and the assembled ZABs. **a**) AC impedance spectra. **b**) ionic conductivity values. **c**) tensile strength of the hydrogel electrolytes. **d**) discharge polarization profiles and corresponding power density curves. **e**) rate performance. **f**) galvanostatic discharge profiles. **g**) GCD cycling curves at 2 mA·cm^{-2}.

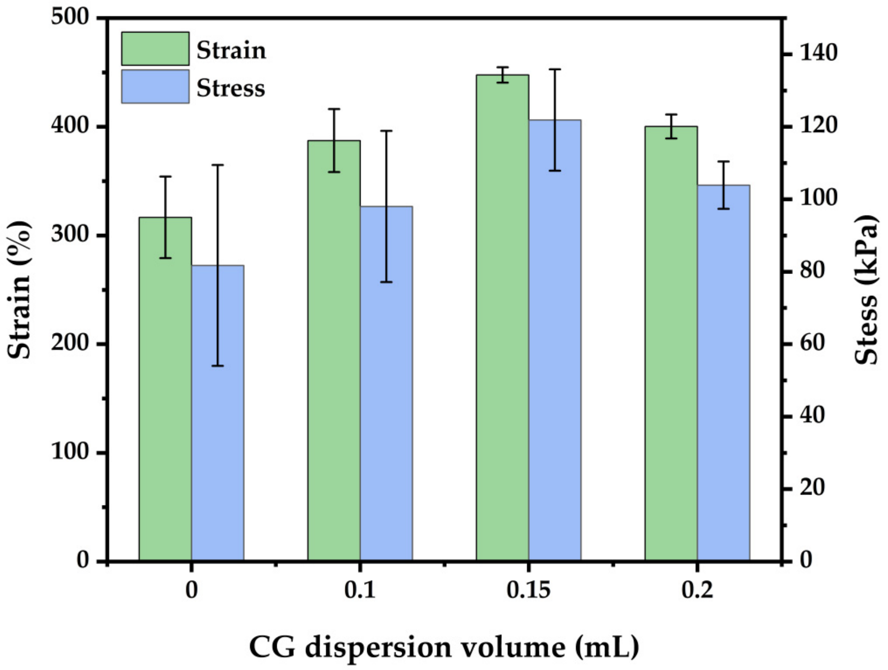

Figure S6. Tensile strain and strength of CNF$_{1\ wt\%}$/PAM and CG@CNF$_{1\ wt\%}$/PAM hydrogel. Data with error bars are presented as mean values ± standard deviations of n = 3 replicates.

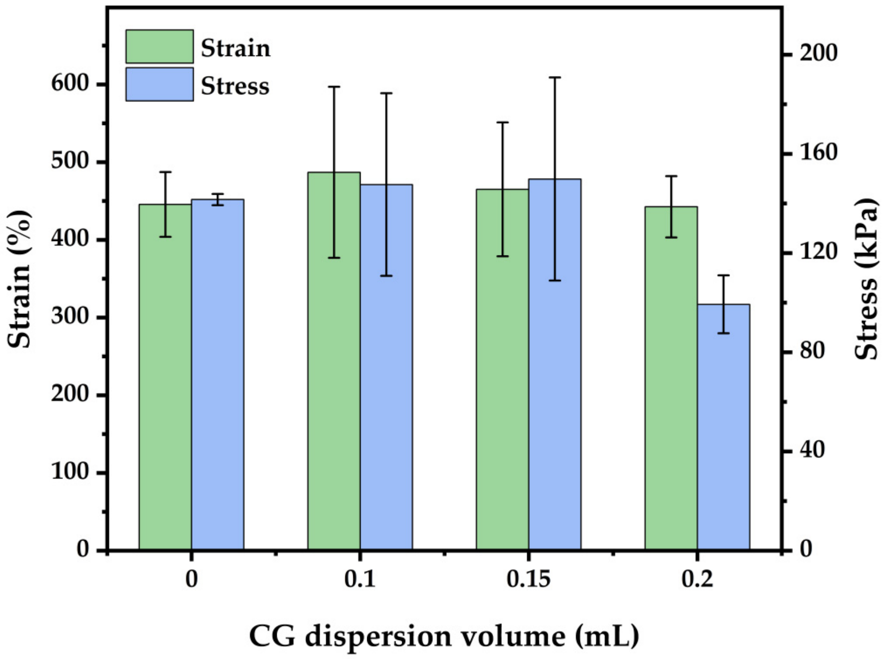

Figure S7. Tensile strain and strength of CNF$_{2\ wt\%}$/PAM and CG@CNF$_{2\ wt\%}$/PAM hydrogel. Data with error bars are presented as mean values ± standard deviations of n = 3 replicates.

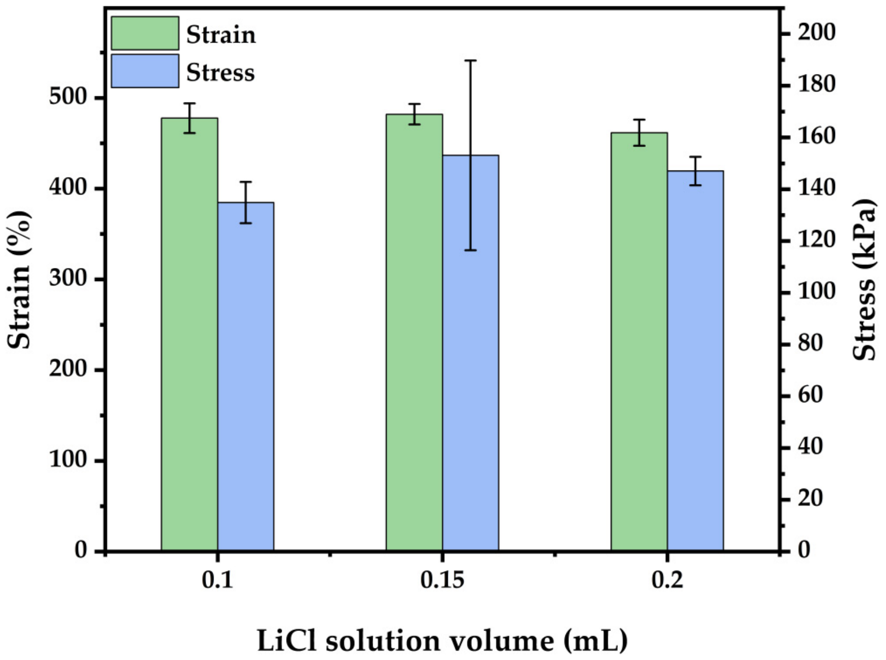

Figure S8. Tensile strain and strength of LiCl-$CG_{0.2}$@$CNF_{2\ wt\%}$/PAM hydrogel. Data with error bars are presented as mean values ± standard deviations of n = 3 replicates.
